# Different anatomic patterns of the indirect tendon of the rectus femoris

**DOI:** 10.1007/s00276-024-03411-z

**Published:** 2024-06-18

**Authors:** S. Mechó, I. Iriarte, R. Lisbona, R. Pérez-Andrés, R. Pruna, A. Rodríguez-Baeza

**Affiliations:** 1 Department of Morphological Sciences (Human Anatomy and Embriology Unit), Faculty of Medicine, Universitat Autònoma de Barcelona, Barcelona, Spain; 2 Department of Radiology, Hospital of Barcelona, Barcelona, Spain; 3 FIFA Medical Center of Excellence, Barcelona Football Club, Barcelona, Spain; 4 Department of Physical Medicine and Rehabilitation, Ars Médica Clinics, Bilbao, Spain; 5 Department of Radiology, Germans Trias i Pujol Hospital, Badalona, Barcelona Spain

**Keywords:** Rectus femoris, Indirect tendon, Anatomic patterns

## Abstract

**Purpose:**

The rectus femoris forms the anterior portion of the quadriceps muscle. It has a proximal tendinous complex, which is constituted by a direct tendon, an indirect tendon, and a variable third tendon. Direct and indirect tendons converge into a common tendon. The purposes of this study are to add anatomical knowledge about the proximal tendinous complex and describe anatomical variants of the indirect tendon and, on these basis, categorize different anatomical patterns.

**Method:**

In this study, 48 hemipelvis from bodies donated to the Universitat Autònoma de Barcelona have been dissected to examine the proximal tendinous complex of the rectus femoris.

**Results:**

The following anatomical variants of the indirect tendon were described: inferior aponeurotic expansion in 23/48 cases (47.9%); superior aponeurotic expansion in 21/48 cases (43.7%); and an unusual origin of the myotendinous junction of the rectus femoris in the free portion of the indirect tendon in 19/48 cases (39.6%). On the basis of the aponeurotic expansions, the following anatomical patterns of the indirect tendon were defined: standard (19/48 cases, 39.6%), superior and inferior complex (15/48 cases, 31.2%), inferior complex (8/48 cases, 16.7%), and superior complex (6/48 cases, 12.5%).

**Conclusion:**

We can categorize four different anatomical patterns of the indirect tendon, three of which are complex. We suggest that complex patterns can cause an increased stiffness of the indirect tendon and so be considered non-modifiable risk factors for rectus femoris injuries. Finally, it would be useful to identify complex patterns and perform injury prevention actions through specific physical preparation programs.

## Introduction

The rectus femoris (RF) forms the anterior layer of the quadriceps and is the sole biarticular head of this muscle. From a phylogenetic perspective, the heads of the quadriceps have become independent and specialized in the evolution from amphibians to mammals [30]. Ontogenetically, the origin comes from a blastematic lamina that covers the anterior aspect of the femoral primordium. This muscular sheet is separated through the highly organized proliferation of connective tissue that occurs late in the development of the limb outline; this process involves hox gene and growth factors, leading to variability in fetal muscle morphology [30].

Division of the RF begins at stage 17 (11–14 mm embryo, 41 days) according to O'Rahilly and Gardner [21]. At stage 20 (18–20 mm embryo, 51 days), the different bellies of the quadriceps muscle are well defined and attached to the skeleton by their definitive tendons [19, 21].

The proximal origin of the RF has been extensively studied from the anatomical point of view [2, 4, 9–11, 15–18, 22, 24, 27, 29]. It consists of a proximal tendinous complex (PTC), which is formed by the direct tendon (DT) and the indirect tendon (IT), that converge into a common tendon (CT). A third tendon originating from the IT has also been described [22]; it is not constantly present, but very frequently (83% in a sample of 96 PTC), according to Tubbs [1, 29]. Finally, a membrane connecting the CT with the anterior superior iliac spine (ASIS) has been recently described as a constant component of the PTC and a possible stabilizer [16].

The DT originates from the anterior inferior iliac spine and the underlying rough surface. It is formed of fibers with a longitudinal craniocaudal direction and a slight medial inclination with respect to the long axis of the muscle. Its myotendinous junction (MTJ) lies very proximal to the thigh, and its tendinous fibrillar structure is distributed along the anterior surface of the muscle, continuing distally with the anterior myoaponeurotic junction and myofascial unit [11, 17, 23, 27]. Its main function is performed at the beginning of the flexion [4].

The IT originates from the supraacetabular sulcus and the lateral aspect of the capsule of the hip joint, resulting in a wide area of origin. It is the largest proximal tendon and develops before the DT; indeed, until the sixth fetal month, only the IT can be distinguished [24, 25, 28]. It has a triangular morphology, follows a posteroanterior course in the axial plane, and usually has a medial inclination with respect to the longitudinal axis of the muscle [2, 9, 13, 18, 19]. It extends along the anterior midline of the muscle, forming the central septum. Then, it thins out and reaches the lower third of the thigh, acquiring a linear shape with a long sagittal axis [9–11, 13–16]. The MTJ of the IT has a longer craniocaudal extension than the DT [15]. The IT performs its main function once the hip flexion has begun [4].

The DT and IT blend in the CT approximately 2 cm from the origin of the DT and 5.5 cm from the origin of the IT. The PTC constitutes a Y-shaped tridimensional structure covered by a common paratendon [2, 4, 7, 9, 10, 12, 13, 15–20, 22, 29].

The third tendon originates from the IT. It follows an inferolateral course forming an approximate angle of 60º with respect to the long axis of the IT, and inserts in the anterior face of the trochanter, between the insertion of the gluteus minimus tendon and the iliofemoral ligament [1, 29]. Armstrong et al. established that the third tendon extends to the posterior MTJ of the central septum [1].

Functionally, the quadriceps muscle is primarily responsible for knee extension, although the RF also performs a certain degree of hip flexion, both movements in the sagittal plane. It stabilizes and guides the knee in different complex movements, as well as the pelvis when standing [9, 15, 17, 24].

In kicking and sprinting sports like football [6], the quadriceps is the second most injured muscle and the RF is the component of the quadriceps that is most frequently injured causing in severe cases longer time loss than hamstring injuries [6, 8, 12]. Muscle injuries risk factors can be divided into intrinsic or extrinsic factors [12]. Intrinsic factors include the anatomy and biomechanics of the muscle and their understanding requires a detailed knowledge of the anatomical characteristics of the RF. In this context, the aim of this study is to investigate the anatomical variants of the PTC of the RF and, on these basis, categorize different anatomical patterns.

## Materials and methods

We studied 48 hemipelvis that included the thigh from bodies donated to the Faculty of Medicine of the Universitat Autónoma de Barcelona (UAB). The average age of the subjects included in the study was 79.7 years (range 54–98). Thirty subjects were females (62.5%) and eighteen were males (37.5%); 45.8% of the limbs were right and 54.2% were left.

Body donation at the UAB is regulated by an acceptance document approved by the Ethics Commission in Animal and Human Experimentation (file CEEAH 2904 of March 11, 2015). All hemipelvis were preserved by arterial perfusion of modified Cambridge solution (phenol, ethanol, glycerin, and formaldehyde) and maintained at 6-7ºC until their use.

The hemipelvis were dissected and examined through a standardized protocol, by planes from the anterior to the posterior aspect of the proximal thigh. The skin and subcutaneous cellular tissue were lifted to identify the sartorius muscle and the femoral (Scarpa’s) and quadriceps triangles. The superficial fascia of the sartorius muscle, and the vessels and nerve contents of the femoral triangle were also removed to expose the iliopsoas muscle. Afterward, the iliopsoas muscle was dissected from the pelvic rim to the extrapelvic portion in the proximal thigh, and the pubic insertion of the inguinal ligament was sectioned, keeping its insertion in the anterosuperior iliac spine undamaged. The connective tissue between the tensor fasciae latae and the sartorius that covers the RF was removed. The origin of the tensor fasciae latae was removed as close as possible to its origin on the ASIS and anterior iliac crest. Then, the gluteal musculature was dissected from the outer ilium to recognize the full extension of the tendinous insertion of the RF.

### Data analysis

The categorical variables were described as absolute frequencies and percentages. The correlations between anatomical variants and demographic variables (gender and laterality) were evaluated using Pearson Chi-Square Test. The level of statistical significance was set to *P* < 0.05, using the statistical software Statplus: Mac Pro Version v7 (AnalySoft Inc) for data analysis.

## Results

**Fig. 1:**
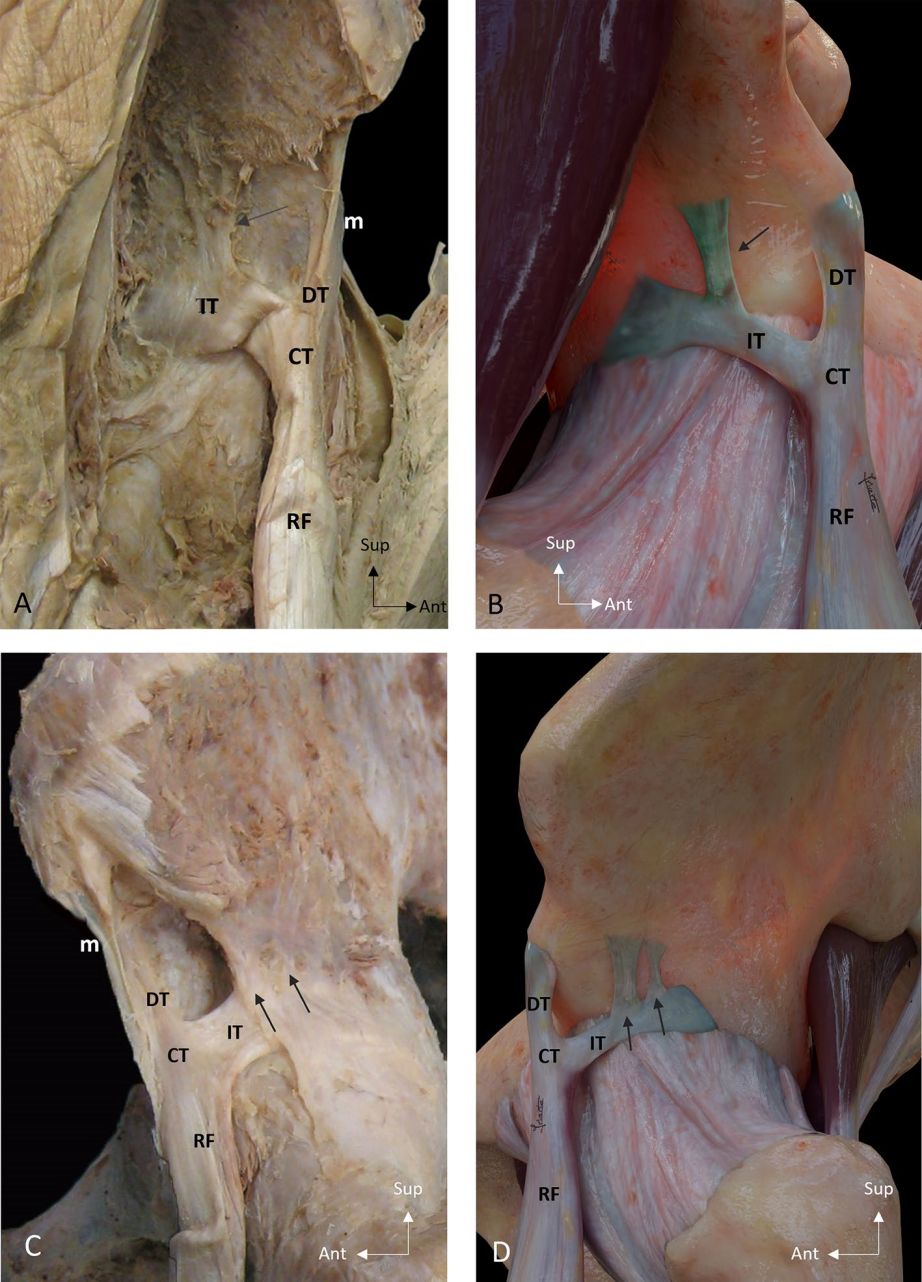
Superior aponeurotic expansion of the indirect tendon. **A** Lateral overview of a right thigh showing a superior aponeurotic expansion (arrow) that connects the superior margin of the indirect tendon (IT) with the acetabular groove. The IT shows a waving morphology. **B** Illustration of a lateral overview of a right thigh showing a superior aponeurotic expansion (arrow). **C** Lateral overview of a left thigh showing two superior aponeurotic expansions (arrows). **D** Illustration of a lateral overview of a left thigh showing two superior aponeurotic expansions. Direct tendon (DT), Common tendon (CT), Membrane common tendon-anterosuperior iliac spine (m), Rectus femoris (RF)

**Fig. 2:**
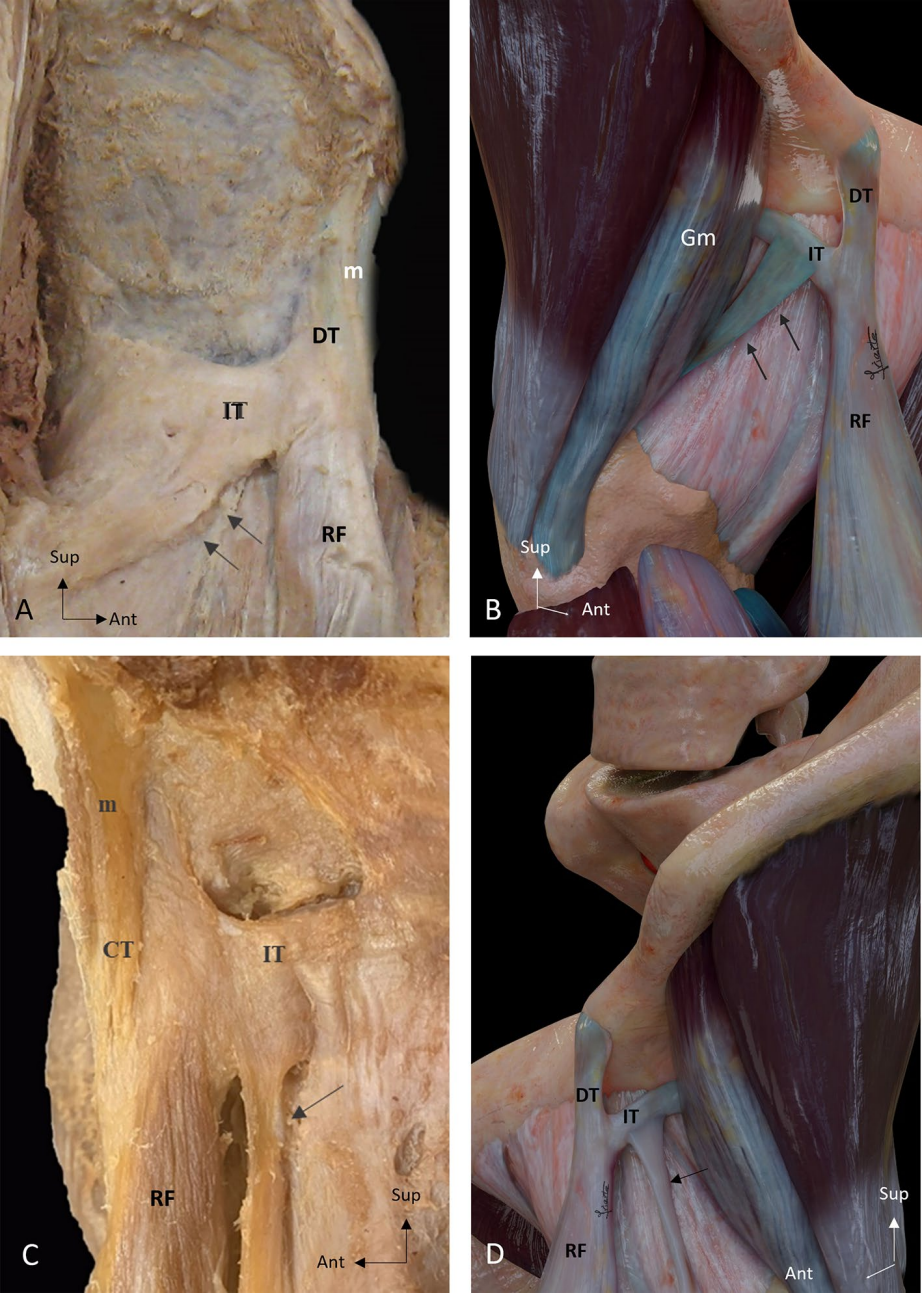
Inferior aponeurotic expansion of the indirect tendon. **A** Lateral overview of a right thigh showing a broad band inferior aponeurotic expansion (arrows) that originates in the inferior margin of the indirect tendon (IT). **B** Illustration of a lateral overview of a right thigh showing a broad band inferior aponeurotic expansion (arrows) that follows an inferior trajectory to communicate with the gluteus minimus (Gm). **C** Lateral overview of a left thigh showing an inferior tendinous expansion (arrow) that expands to an accessory fibrous tract [16]. **D** Illustration of an anterolateral overview of a left thigh showing the inferior tendinous expansion (arrow) Direct tendon (DT), Common tendon (CT), Membrane common tendon-anterosuperior iliac spine (m), Rectus femoris (RF)

**Fig. 3:**
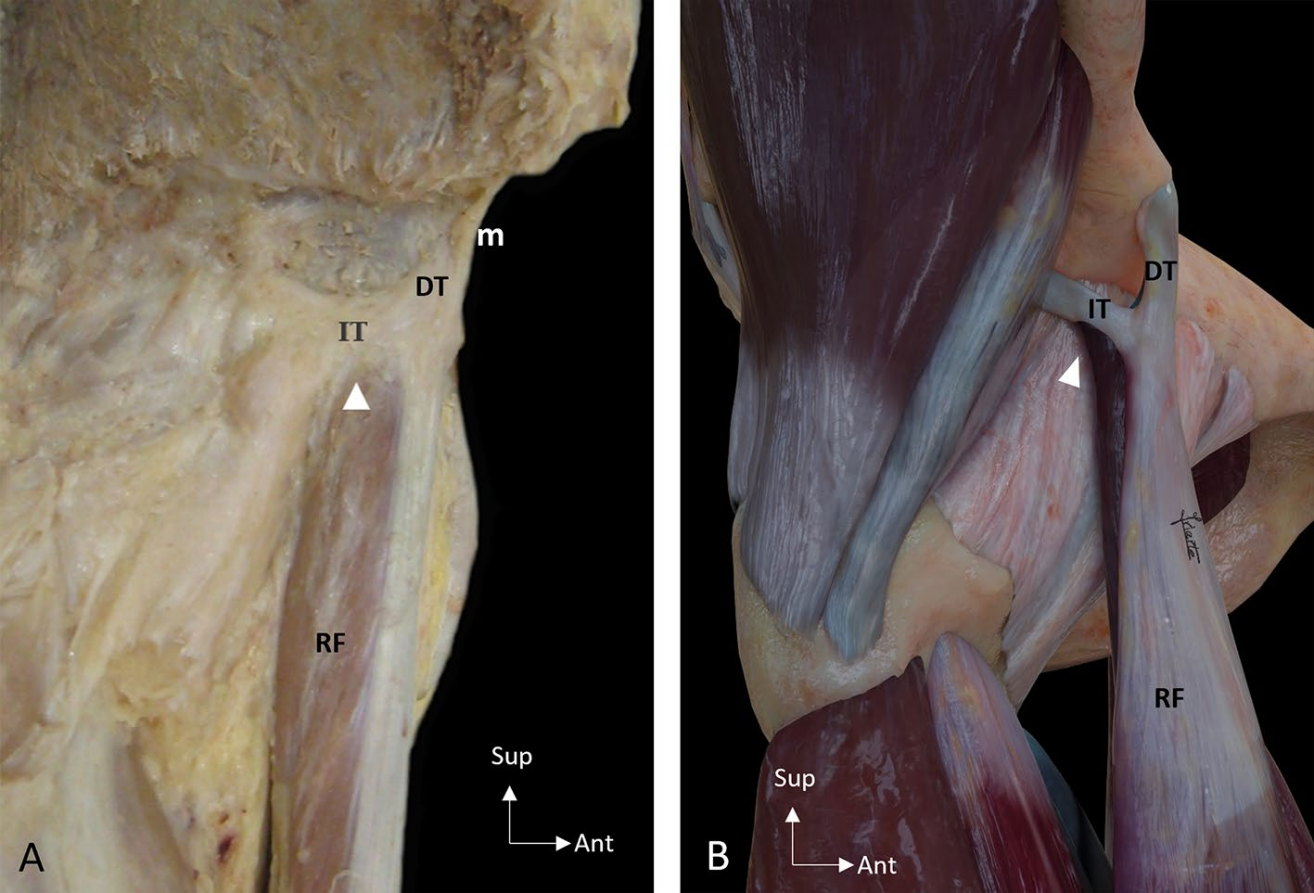
Unusual origin of the myotendinous junction of the rectus femoris (arrowhead). **A** Lateral overview of a right thigh showing the unusual origin of muscle fibers from the indirect tendon (IT). **B** Illustration of an anterolateral overview of a right thigh showing the unusual origin of muscle fibers. Direct tendon (DT), Membrane common tendon-anterosuperior iliac spine (m), Rectus femoris (RF)

In 19 cases of the 48 hemipelvis studied, no anatomical variant was found. In the remaining 29 cases different anatomical variants of the PTC of the RF were identified:

1. *Superior aponeurotic expansion of the IT (21/48 cases, 43.7%)* It follows a vertical path and connects the superior margin of the IT in its anterior or middle third with the acetabular groove. In six cases (6/21, 28.6%), there was more than one superior expansion (Fig. 1).
2. *Inferior aponeurotic expansion of the IT (23/48 cases, 47.9%)* It was the most frequent anatomical variant. It corresponds with the third tendon that originates from the inferior margin of the IT and extends caudally between the gluteus minimum and the ilifemoral ligament. It can present a tendinous morphology (4/23 cases, 17.4%) or a broad band (19/23 cases, 82.6%) (Fig. 2).
3. *Unusual origin of the MTJ of the RF (19/48 cases, 39.6%)* It is characterized by the origin of muscle fibers of the RF in the free portion of the IT, prior to the formation of the CT (Fig. 3).

**Fig. 4:**
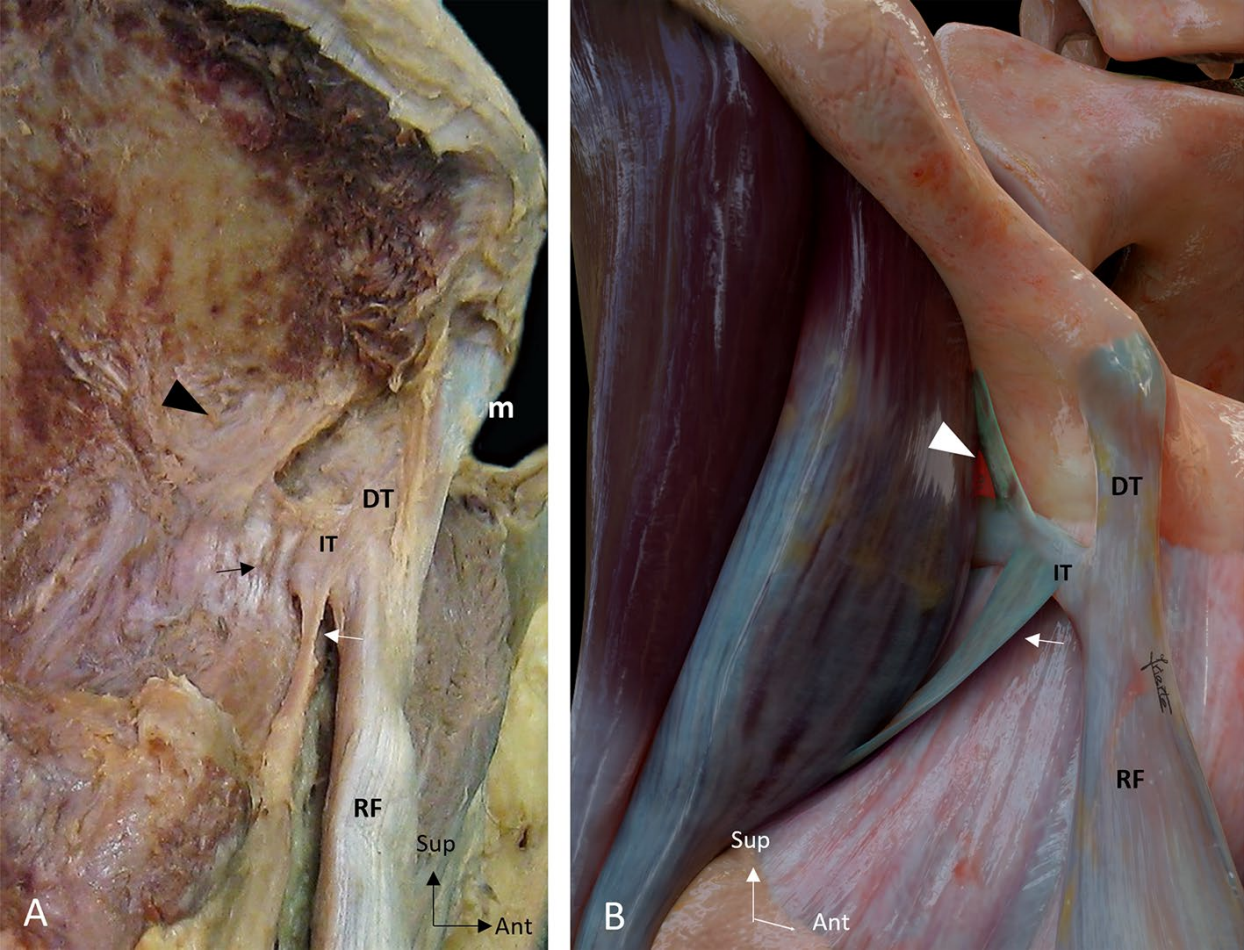
Superior and inferior complex indirect tendon. **A** Lateral overview of a right thigh showing a superior and inferior complex indirect tendon (IT), with wide superior aponeurotic expansion (arrowhead) and inferior tendinous expansion (white arrow). Pay attention to the waving morphology of the IT (black arrow). **B** Illustration of a lateral overview of a right thigh showing the proximal tendinous complex of the rectus femoris with a superior and inferior complex IT. Direct tendon (DT), Membrane common tendon-anterosuperior iliac spine (m), Rectus femoris (RF)

On the basis of the aponeurotic expansions, we identified four IT anatomical categories:

1. *Standard IT*, with no aponeurotic expansions, in 19/48 cases (39.6%)
2. *Superior and inferior complex IT*, with superior and inferior aponeurotic expansions, are in 15/48 cases (31.2%) (Fig. 4)
3. *Inferior complex IT*, with inferior aponeurotic expansions, in 8/48 cases (16.7%)
4. *Superior complex IT*, with superior aponeurotic expansions, in 6/48 cases (12.5%).

**Fig. 5:**
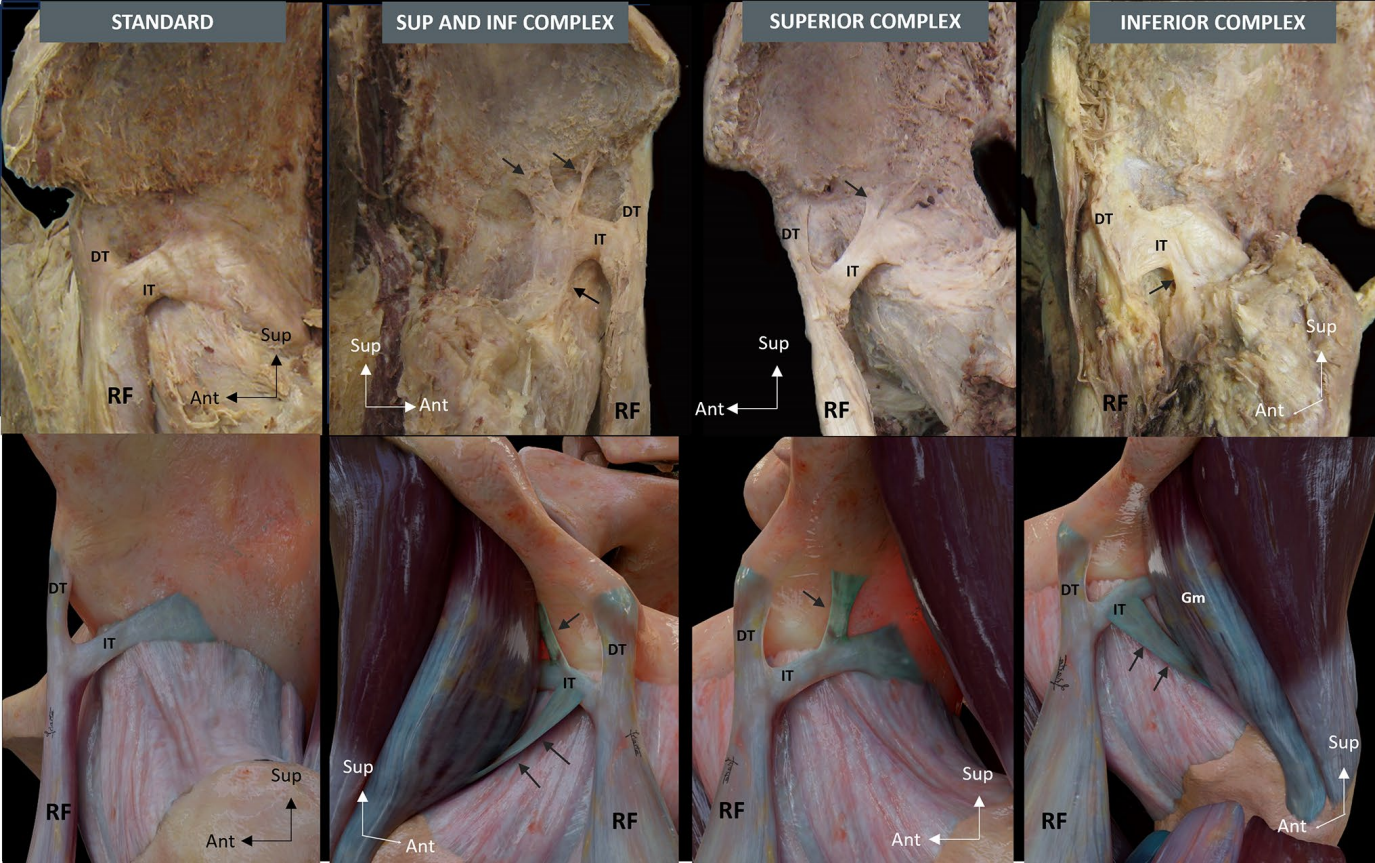
Indirect tendon morphological categories (figures and illustrations of each type). Superior and inferior expansions (arrows). Indirect tendon (IT), Direct tendon (DT), Rectus femoris (RF), Gluteus minimus (Gm)

In Fig. 5 there are all IT anatomical categories with the illustration of each type.

In Table 1 we show the distribution of the anatomical variants depending on gender and laterality. We found no correlation between the different variants and demographic factors (Table 2).

**Table 1:** Distribution of anatomical variants according to gender and laterality (absolute frequencies and percentages)

	Superior expansion IT	Inferior expansion IT	Unusual MTJ
Female	13 (61.9%)	15 (65.2%)	11 (57.9%)
Male	8 (38.1%)	8 (34.8%)	8 (42.1%)
Left	9 (42.8%)	13 (56.5%)	10 (52.6%)
Right	12 (57.2%)	10 (43.5%)	9 (47.4%)

**Table 2:** Statistical results of Pearson chi-square test analyzing the correlation of the different anatomical variants with gender and laterality

	Superior expansion IT	Inferior expansion IT	Unusual MTJ
Gender	0.25	0.68	0.67
Laterality	0.37	0.49	0.85

P values

## Discussion

Muscle lesions are the most common injuries in athletes, and they represent more than 30% of the injuries in soccer players; 79% occur in the thigh, and 19% in the quadriceps [6, 8, 11]. In particular, the RF is the component of the quadriceps that is more frequently injured in sports involving repetitive kicking and sprinting [4, 15–17, 24, 29]. Different studies monitored these lesions in Australian footballers and showed that they are associated with a long time to rehabilitate and delayed return-to-play [3, 5, 17]. Therefore, from a clinical point of view, we are interested in studying all the elements of the RF muscle, its anatomical variants, and the relationships between them.

Several anatomical variants have been described at the origin of the RF: two tendons originating from the anteroinferior iliac spine, a continuity between the DT and the IT, and the absence of IT [14]. Astzmon et al. performed one of the most complete studies on the anatomy of the IT [2]. However, neither in this publication nor in any other there is a description of superior aponeurotic expansions related to the IT, or an unusual origin of the MTJ in the IT.

In this study, anatomical variants of the PTC of the RF were identified in 29 of 48 cadavers (60.4%). The most common one consisted in inferior aponeurotic expansions of the IT, followed by superior aponeurotic expansions of the IT, and unusual origins of the MTJ in the free portion of the IT. Among these. we observed some thicker-than-usual inferior aponeurotic expansions that could be considered third tendons according to Tubbs and Paturet description [1, 22, 29]. The remainder of the inferior expansions consist of broad bands in the anteroposterior axis that course in close relation to the capsular iliofemoral ligament and suggest a capsular fascial reinforcement. All these aponeurotic expansions are connective tissue and belong to “the fascial system” according to the Fascia Nomenclature Committee [26].

The IT performs its main function once the hip flexion has begun [4]. At rest, in the supine position and standing, the IT has a wavy morphology (Fig. 1A, 2C, and 4A). We think that the IT needs a certain degree of mobility. In the case of complex morphology, we speculate that the IT would be more rigid, the mobility would decrease, and the risk of injury would increase. The aponeurotic expansions could cause greater stiffness, while the unusual MTJ might not, only entailing a variation in the transmission of forces of the PTC. Therefore, the unusual MTJ was not included in our classification of the anatomical patterns.

As anatomical predispositions, complex patterns can be considered non-modifiable risk factors for RF injuries [12, 17]; however, detecting these predispositions allows applying early physical preparation programs to prevent injuries.

A limitation of this study is the sample size including only one hemipelvis per cadaver. Future studies must assess both hemipelves in each to investigate whether the IT category is bilaterally equal. Another limitation is that we did not include the entire lower extremity; therefore, we can speculate on the biomechanics of the complex patterns.

To confirm the greater stiffness of complex anatomical patterns and its relationship with the risk of injury to the proximal MTJ of the RF, we propose to perform further studies by diagnostic imaging (MRI or US).

The main novelty of this study is the description of superior and inferior aponeurotic expansions of the IT that led to its classification as standard, superior and inferior complex, inferior complex, and superior complex. We speculate that complex patterns could affect the function of the IT and be considered non-modifiable risk factors for RF injuries [12, 17]; therefore, detecting complex patterns would allow applying early physical preparation programs to prevent injuries.

## Conclusions

We describe new aponeurotic expansions to the IT. On the basis of their presence, we define four anatomical patterns of the IT, three of which are complex, possibly leading to greater IT stiffness. Therefore, complex anatomical IT patterns could be non-modifiable intrinsic risk factors for RF injuries. We believe that specialists in sports medicine, above all radiologists, should be aware of these anatomical categories to detect athletes who could benefit from a primary preventive plan for injuries to the proximal MTJ of the RF.

## Data Availability

Any datasets used in this study can be accessed.
